# Periaqueductal grey cyclooxygenase-dependent facilitation of C-nociceptive drive and encoding in dorsal horn neurons in the rat

**DOI:** 10.1113/jphysiol.2014.275909

**Published:** 2014-10-20

**Authors:** J Lianne Leith, Alex W Wilson, Hao-Jun You, Bridget M Lumb, Lucy F Donaldson

**Affiliations:** 1 School of Physiology & Pharmacology, University of BristolBristol, BS8 1TD, UK; 2 Neurosciences CEDD, GlaxoSmithKline, HarlowEssex, CM19 5AW, UK; 3 Center for Biomedical Research on Pain (CBRP), College of Medicine, Xi'an Jiaotong UniversityXi'an, 710061, PR China; 4 Arthritis Research UK Pain Centre, School of Life Sciences, QMC, University of NottinghamNottingham, NG7 2UH, UK

## Abstract

**Abstract:**

The experience of pain is strongly affected by descending control systems originating in the brainstem ventrolateral periaqueductal grey (VL-PAG), which control the spinal processing of nociceptive information. A- and C-fibre nociceptors detect noxious stimulation, and have distinct and independent contributions to both the perception of pain quality (fast and slow pain, respectively) and the development of chronic pain. Evidence suggests a separation in the central processing of information arising from A- *vs*. C-nociceptors; for example, inhibition of the cyclooxygenase-1 (COX-1)–prostaglandin system within the VL-PAG alters spinal nociceptive reflexes evoked by C-nociceptor input *in vivo* via descending pathways, leaving A-nociceptor-evoked reflexes largely unaffected. As the spinal neuronal mechanisms underlying these different responses remain unknown, we determined the effect of inhibition of VL-PAG COX-1 on dorsal horn wide dynamic-range neurons evoked by C- *vs*. A-nociceptor activation. Inhibition of VL-PAG COX-1 in anaesthetised rats increased firing thresholds of lamina IV–V wide dynamic-range dorsal horn neurons in response to both A- and C-nociceptor stimulation. Importantly, wide dynamic-range dorsal horn neurons continued to faithfully encode A-nociceptive information, even after VL-PAG COX-1 inhibition, whereas the encoding of C-nociceptor information by wide dynamic-range spinal neurons was significantly disrupted. Dorsal horn neurons with stronger C-nociceptor input were affected by COX-1 inhibition to a greater extent than those with weak C-fibre input. These data show that the gain and contrast of C-nociceptive information processed in individual wide dynamic-range dorsal horn neurons is modulated by prostanergic descending control mechanisms in the VL-PAG.

## Introduction

Descending control from the periaqueductal grey (PAG) dynamically modulates spinal nociceptive processing as part of coordinated behavioural responses (Bandler *et al*. 2000; Lovick & Bandler, 2005). Although descending control systems from PAG were considered to be solely inhibitory, it is now clear that facilitatory influences can also be evoked from the PAG (Heinricher *et al*. 2004; Martins *et al*. 2010; Chu *et al*. 2012). The balance of descending inhibition and facilitation is critical in determining the output from the dorsal horn, and the resultant sensory, autonomic and behavioural consequences of nociceptive input (Lovick & Bandler, 2005; Heinricher *et al*. 2009).

Descending controls from the PAG discriminate between information originating from A- and C-fibre nociceptors, preferentially controlling C-fibre-evoked nociceptive processing (Waters & Lumb, 1997; McMullan & Lumb, 2006a,*b*2006b; Koutsikou *et al*. 2007). Cyclooxygenase-1 (COX-1) inhibitor and prostaglandin administration into the ventrolateral (VL-) PAG preferentially inhibit or facilitate C-fibre-evoked withdrawal reflexes, respectively (Leith *et al*. 2007).

The acute pain experience evoked by C-nociceptor activation is very different from A-nociceptor activation (slow, burning, poorly localised *vs*. sharp, well localised (Ochoa & Torebjork, 1989; Magerl *et al*. 2001)). The nociceptor sub-types also play distinct roles in chronic pain states (Fuchs *et al*. 2000; Magerl *et al*. 2001). Increased C-nociceptor activity, associated with tissue damage, initiates and maintains central sensitisation, which underpins the cardinal signs of chronic pain, such as secondary hyperalgesia and allodynia. Importantly, C-nociceptor drive is necessary for spinal sensitisation to A-fibre inputs (Treede & Magerl, 2000; You *et al*. 2010). The preferential modulation of C-nociceptive input by the PAG therefore has important functional consequences. It is now apparent that pathological alterations in descending control systems also contribute to the development and maintenance of chronic pain states (Vanegas & Schaible, 2004; Pertovaara, 2006; Saade & Jabbur, 2008).

The COX–prostaglandin system within the PAG plays an important role in regulating spinal nociceptive processing. COX-1, COX-2 and receptors for prostaglandin E2 (PGE2) are expressed in the PAG (Breder *et al*. 1992, 1995; Matsumura *et al*. 1992; Ek *et al*. 2000; Nakamura *et al*. 2000). Prostaglandins or synthetic analogues inhibit glutamatergic (Lu *et al*. 2007) and potentiate GABAergic and glycinergic transmission in the PAG (Vaughan, 1998; Shin *et al*. 2003). *In vivo*, PAG COX-1 facilitates spinal nociceptive withdrawal reflexes (Leith *et al*. 2007), and microinjection of PGE2 into VL-PAG produces hyperalgesia (Heinricher *et al*. 2004; Oliva *et al*. 2006; Leith *et al*. 2007). Functional PGE2 receptors in the PAG facilitate both acute chemical and chronic neuropathic pain (Oliva *et al*. 2006; Palazzo *et al*. 2011). Although administration of non-specific COX inhibitors into the PAG alters dorsal horn neuronal activity (Carlsson *et al*. 1986; Carlsson & Jurna, 1987; Vanegas *et al*. 1997; Hernandez & Vanegas, 2001), the mechanisms through which the COX-1-dependent facilitatory system from PAG to spinal cord modulates spinal sensory processing remain largely unknown. We therefore determined the actions of the descending control pathways regulated by COX-1 activity in the (VL)-PAG on their sensory neuronal targets in the spinal dorsal horn, to provide further insights into the neuronal mechanisms underlying differential control of A- and C-fibre-evoked spinal nociception. A novel aspect of the study was to quantify, and then relate, the degree of descending tonic facilitation to the magnitude of C-fibre drive to dorsal horn neurons. C-nociceptors have a key function in the establishment of central sensitisation, generating central neuronal changes underpinning the establishment of chronic pain states (Stemkowski & Smith, 2012). A-nociceptors signal acute pain and serve a protective function under normal conditions (Cervero, 2009), whereas C-nociceptor-driven central sensitisation facilitates processing of A-nociceptor inputs in pain states (Magerl *et al*. 2001). Given these distinct roles of A- and C-nociceptors in physiological and pathophysiological nociceptive processing, understanding the dorsal horn neuronal mechanisms underlying the preferential control of these different functional spinal inputs is highly clinically relevant.

## Methods

All experiments were carried out in accordance with the UK Animals (Scientific Procedures) Act, 1986 and associated guidelines, and with approval of the Ethical Review Groups of the University of Bristol and GSK. A total of 46 male adult Wistar rats (280–320 g) were used in these experiments. Animals were housed in standard conditions and handled frequently to minimise stress on the day of the experiment.

### Animal preparation – behavioural study

Twenty rats were used in these experiments. Animals were anaesthetised with 2–4% isofluorane in oxygen. The head was secured in a stereotaxic frame and a stainless steel intracerebral guide cannula (26 gauge, cannula cut 8 mm below the pedestal; PlasticsOne, Voanoke, VA, USA) was implanted in the left VL-PAG (coordinates relative to Bregma 7.4–7.5 caudal, 0.9 mm left of midline, 6.5 mm below skull surface) and secured to the skull with screws and cyanoacrylate (RS Components, Corby, UK). A stylet was secured in the cannula to maintain patency. Animals received antibiotic cover, 1 ml kg^−1^ Synulox s.c. (clavulanic acid 35 mg ml^−1^ and amoxycillin 140 mg ml^−1^, Pfizer UK), and analgesia, 0.4 ml Rimadyl s.c. (carprofen 50 mg ml^−1^) during surgery. Following a recovery period of at least 5 days, three baseline paw withdrawal thresholds to a thermal ramp stimulus were measured (22–50°C ramp at 1°C s^−1^ delivered using a Peltier device to the left hind paw; equipment built in-house). The temperature at which the paw was withdrawn from the thermal stimulus was taken as the end point. A minimum of 30 min was left between repeated thermal stimuli on the same animal.

Animals were assigned randomly to one of two experimental groups (each *n* = 10) for drug administration. Compounds were administered via the implanted guide cannula, vehicle (300 nl 30% DMSO in physiological saline) or ketoprofen (COX inhibitor; 10 μg in 300 nl) as used previously in terminally anaesthetised animals (Leith *et al*. 2007). The experimenter was blinded to the identity of the drug administered during the testing phase. Compounds were injected into the PAG using an internal ‘injector’ guide cannula cut to project 0.5 mm beyond the end of the implanted guide cannula (PlasticsOne) connected to a 1 μl syringe (Scientific Glass Engineering, Ringwood, Vic., Australia). Animals were held securely and the stylet removed from the implanted guide cannula. Compounds were injected over 1 min and the injector was left in place for an additional minute after the completion of the injection to prevent backflow of the compound up the cannula. The stylet was then replaced into the implanted cannula. Paw withdrawal thresholds to the thermal ramp device were tested again 30 min after drug administration. At the end of the behavioural experiments, animals were killed by a rising concentration of carbon dioxide. Brains were removed and fixed in 4% paraformaldehyde in 0.1 m phosphate buffer for at least 24 h, then cryoprotected in 30% sucrose solution for at least 24 h, before sectioning at 60 μm. PAG injection sites were localised with reference to a rat brain atlas (Paxinos & Watson, 2006). Animals in which the cannula was found to have been outwith the VL-PAG were excluded from further analysis. As a result of *post hoc* injection site analysis, two animals were removed from the ketoprofen group, and four from the vehicle group in the behavioural experiments.

### Animal preparation terminal experiments

Twenty-six rats were used in these experiments. Anaesthesia was induced using 4% halothane in O_2_ delivered through a mask over the snout, and a branch of the external jugular vein was cannulated for anaesthetic maintenance. A branch of the carotid artery was exposed and cannulated for recording of blood pressure. Body temperature was maintained within physiological limits by means of a feedback-controlled heating blanket and rectal probe. A laminectomy was carried out between T11 and T13 to record from lumbar dorsal horn neurons. Animals were then positioned in a stereotaxic frame and a craniotomy was performed, to allow micropipette access to the PAG. Following surgery, anaesthesia was maintained using a constant i.v. infusion of alphadolone/alphaxalone (∼20 mg kg^−1^ h^−1^; Saffan, Schering Plough Animal Health, UK) at a level at which there were no precipitous changes in blood pressure to minor noxious stimuli.

### Preferential activation of A- or C-nociceptors

A- or C-heat nociceptors were stimulated using a custom made heating lamp system to deliver fast or slow rates of skin heating respectively to the dorsal surface of the hindpaw, as described previously (McMullan *et al*. 2004; Leith *et al*. 2007). In brief, heat from a sputter-coated projector bulb was focused onto a blackened copper disc positioned at the focal point. A T-type thermocouple (made in-house: 0.02 mm diameter copper/constantin) was fixed to the outer surface of the copper plate which measured the surface temperature of the skin when placed in firm, even contact with the hindpaw dorsum. Using a constant bulb voltage, fast rates of heating (contact heating rate 7.5 ± 1°C s^−1^) were used to preferentially activate A-fibre (myelinated, capsaicin-insensitive) heat nociceptors, whereas slow rates of heating (contact heating rate 2.5 ± 1°C s^−1^) were used to preferentially activate C-fibre (unmyelinated, capsaicin-sensitive) heat nociceptors. Our previous studies (McMullan *et al*. 2004) demonstrate that these heating rates measured at the skin surface reliably reproduce the same subcutaneous heating rates as those previously described to preferentially activate A- *vs*. C-nociceptors (Yeomans *et al*. 1996; Yeomans & Proudfit, 1996), and that they are not adversely affected by alterations in local blood flow resulting from mild inflammation (Drake *et al*. 2014). The cut-off temperature of the heat lamp was controlled via a Spike2 script to prevent tissue damage. Fast or slow heat ramps were carried out at 8 min intervals to avoid sensitisation (McMullan *et al*. 2004).

### Recording of dorsal horn neuronal activity

The vertebral column was clamped at each end of the laminectomy to maximise the stability of the preparation. The dura was removed, a pool was made with skin flaps and the whole area was filled with agar to further stabilise the preparation. Once set, a small window was cut out of the agar over the desired recording site and filled with warm paraffin oil. A glass-coated tungsten microelectrode (∼5 MΩ; Merrill & Ainsworth, 1972) was lowered into the cord at the rostrocaudal location at which maximum field potentials had been observed to low intensity percutaneous electrical stimulation of the hairy skin of the contralateral hindpaw. Single-unit neuronal activity was amplified (×5000) and filtered (500 Hz–10 KHz; Neurolog system) before being captured for subsequent analysis via a 1401plus (CED, Cambridge, UK) onto a PC running Spike2 v5 software (CED).

### Functional classification of spinal neurons

Wide dynamic-range (WDR; class 2) cells in the deep dorsal horn were located using low threshold search stimuli (gentle tapping or brushing) delivered to the ipsilateral (left) hindpaw. Responsive cells were then tested with high threshold, noxious stimuli (firm pinch with untoothed forceps, and fast and slow heat ramps, to preferentially activate A- or C-heat nociceptors, respectively, as above). Only cells that responded to both low and high threshold stimuli (including heat) were characterised further, corresponding to the class 2 neurons described previously (Menetrey *et al*. 1977, 1979). Once a suitable cell was located, its excitatory receptive field (ERF) was mapped using both low and high threshold stimuli (brush and firm pinch, respectively). Only cells that had ERFs located on the dorsal surface of the left hindpaw that were both accessible to, and larger than, the copper disc of the heating lamp were included in this study. It was not possible to investigate the responses of class 3 neurons in this study as a low threshold search stimulus was used. Prolonged use of high intensity electrical stimulation that would be necessary to locate class 3 units (Light *et al*. 1986) was considered unsuitable due to the risc of high intensity stimuli evoking changes in normal somatosensory processing. Deep dorsal horn neurons were studied here because although superficial (e.g. lamina I) neurons are also important in the processing of nociceptive inputs, deep dorsal horn neurons integrate A- and C-nociceptive inputs, enabling study of responses to both nociceptor types, and in general give larger responses to nociceptive inputs than lamina I neurons (Seagrove *et al*. 2004).

Neuronal activity of class 2 neurons was recorded continuously at 10^3^ samples s^−1^ using Spike2 software. A ‘wavemark’ template was created so that all matching spikes from the same cell were written to a separate channel at 20,000 samples s^−1^. This channel was duplicated and displayed both as the waveform of each event and as firing frequency, from which the rate of spontaneous activity and the responses of the cell to peripheral stimuli could then be measured.

Previous work from this laboratory suggests that the effects of PAG modulation on noxious pinch-evoked activity in dorsal horn neurons might be related to the profile of afferent input to the neuron under study (Waters & Lumb, 2008). Therefore, the afferent input to the cell was characterised by percutaneous electrical stimulation at the centre of the ERF, i.e. the site at which the largest response was evoked (as determined by responses to high and low intensity stimuli as described above) via needle electrodes placed 5 mm apart and delivering constant voltage square pulses at 0.1 Hz, 1 ms duration (DS2 isolated stimulator; Digitimer, Welwyn Garden City, UK). Electrical thresholds for activation of A- and C-fibres were determined by the appearance of a short (<20 ms) and long (90–350 ms; Urch & Dickenson, 2003) latency response, respectively. Cells that responded with one or more spikes at C-fibre latency were classified as C+ve (Waters & Lumb, 2008). To quantify C-fibre-evoked activity, repeated electrical stimuli were delivered at both 1.5 and 3 times C-fibre threshold voltage in most cases. In four cells, repeated sweeps were made only at 1.5 times C-fibre threshold and in four cells only at 3 times C-fibre threshold. Peristimulus time histograms and/or raster plots were constructed from 20 sweeps at 1.5 and 3 times C-fibre threshold, and the response (mean spikes per sweep) of each cell at C-fibre latency was determined.

Responses to slow and fast heat ramps were quantified by dividing the total spike number by the ramp length (giving spikes s^−1^) and corrected for spontaneous activity (SA). SA was measured over a 10 s period prior to ramp onset excluding the period immediately prior to each ramp as removal of a foil barrier in the lamp apparatus at the start of each ramp sometimes elicited a short mechanically evoked burst of firing in some cells. Correlations of response to slow ramps *vs*. spikes at C-fibre latency were made for both 1.5 and 3 times C-fibre threshold. The population of cells included in this study is the same as that previously presented (see Fig.2; Leith *et al*. 2007), in which some of the peripheral characteristics of the population were characterised. These data are, however, not re-presented here.

### Experimental protocol

The effect of administration of the COX-1 inhibitor SC-560 or its vehicle (PBS, Sigma, Poole UK) into the VL-PAG was tested in 18 of the 27 cells, on neuronal responses to fast and slow heat ramps applied to the centre of the ERF. The data from the nine other cells had been previously collected from naïve animals (*n* = 9) that were used to generate baseline data on spinal dorsal horn responses evoked by fast and slow heat ramps. The effect of injection of drug or vehicle on responses to both fast and slow heat ramps in individual animals was tested in 12 cells (6 with SC-560, 6 with vehicle). In these experiments, fast and slow heat ramps were alternated over the course of the experiment. In one of the 6 vehicle cells, it was possible to make a second injection and the effects of SC-560 on slow heat ramps were also tested. In the remaining cells, the effect of SC-560 on either fast (*n* = 4) or slow (*n* = 2) heat ramps was determined. Thus, in the 18 cells, in a subset of 12 animals, 9 cells were treated with SC-560 and tested with slow ramps, and 10 were treated with SC-560 and tested with fast ramps. The remaining six animals were treated with vehicle and both fast and slow ramps. For analysis, in the cells tested with both fast and slow ramp responses, each was treated as an independent data point.

Once reproducible baseline values of neuronal responses to fast or slow heat ramps were achieved, a glass micropipette containing drug (SC-560, 300 nl, 50 nm; Smith *et al*. 1998; Leith *et al*. 2007) or vehicle (0.0005% DMSO in saline) solution was lowered vertically into the VL-PAG contralateral to the stimulated hindpaw.

Glass micropipettes contained SC-560 or vehicle plus 5% Pontamine Sky Blue dye to mark injection sites. Final drug doses used were based on effective doses as described and previously used (Smith *et al*. 1998; Leith *et al*. 2007). Solutions were pressure ejected from the micropipette over 1 min (starting at *t* = 0 min as illustrated in the graphs). Fast or slow heat ramps were resumed at 1 min and neuronal activity was recorded for a further 65 min.

The depth of neuronal recordings was measured from the surface of the spinal cord using a calibrated depth display on the stepping micro-drive used to position the electrode for recording, and visually verified at the correct depth using Pontamine Sky Blue injections at the surface, and 500 μm and 1500 μm at the end of an experiment.

### Data analysis

Quantification of electrically evoked afferent input and of responses to fast and slow heat ramps was carried out as described above. Neuronal responses to fast and slow heat ramps were then analysed in greater detail with respect to the firing threshold and suprathreshold activity as reported previously (McMullan & Lumb, 2006b). In brief, because many cells exhibited some level of SA, threshold was defined as the temperature at which firing exceeded SA (by 2 SD) and remained elevated above SA throughout the remainder of the ramp.

### Effects of SC-560 or vehicle administration into the VL-PAG on dorsal horn neuronal responses to fast and slow heat ramps

For each cell, both the peak change in threshold post-SC-560 or vehicle (the time point at which the firing threshold post-SC-560 or vehicle was most altered (either positively or negatively) from control) and the overall effect over time on thresholds was measured (the area under the curve (AUC) of the graph of threshold against time, over the period 0–65 min after drug administration).

The ability of dorsal horn neurons to encode temperature changes during heat ramps in their firing patterns was also analysed, as previously described (McMullan & Lumb, 2006b). Temperature encoding was calculated for control (pre-SC-560) responses and also at the time point post-SC-560 at which the activity of each cell was most altered (peak positive or negative) from control. Briefly, a Spike2 script (written by S. Gray, CED) generated bins for every 1°C above threshold and calculated spike counts for each bin (spikes °C^−1^ per bin) and the width of each bin (s). Spikes °C^−1^ per bin was corrected for bin width giving spikes s^−1^ °C^−1^, then corrected for SA. Three control data sets from each neuron were averaged and the mean data set normalised against the first bin of the response (i.e. [bin *X* response /bin 1 response] × 100% = normalised response; therefore, first bin of response = 100%). For post-SC-560 responses, the raw data set was normalised against the first bin of the baseline response.

At the end of experiments, animals were killed by an overdose of sodium pentobarbital (30 mg i.v. bolus; Sigma) and injection sites were localised as detailed for the behavioural experiments above. None of the animals in terminal experiments was excluded after injection site analysis.

### Statistical analyses

For statistical comparison of outcome measures using Graphpad Prism versions 4–6, normally distributed data with equal variance were compared using Student's *t*-tests (two groups, paired or unpaired as appropriate, e.g. Fig.2) or one-way ANOVA (three or more groups, e.g. Fig.1) as detailed in the text. If the data did not meet the assumptions of parametric tests (non-Gaussian, unequal variances), then non-parametric test equivalents were used, as stated in the text and figure legends. Correlations were tested using Spearman's correlation analysis. Encoding functions were generated by regression analyses of the data (linear or non-linear). Curves were fit using a least squares fit. Comparison of whether linear or Gaussian fits best fit the data sets, or whether a single curve (either linear or Gaussian) fit both data sets before and after PAG drug or vehicle delivery was made using an extra-sum-of-squares F test in Prism 6. Effects of drug compared to vehicle over time were compared using two-way ANOVA, with the assumptions associated with Gaussian distributions.

**Figure 1:**
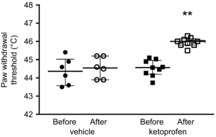
The effect on thermal paw withdrawal thresholds of acute administration of the COX inhibitor ketoprofen into the PAG, in the awake animal Data are shown as individual points, lines are medians and interquartile ranges before (filled) and after (white/crosshatched) ketoprofen (*n* = 8) or vehicle (*n* = 6) administration into the PAG (Kruskal–Wallis statistic = 18.45, *P* = 0.0004 followed by *post hoc* Dunn's tests, **P < 0.01, compared to both before ketoprofen and after vehicle).

## Results

### Are descending prostanergic facilitatory controls tonically active or evoked by experimental surgery?

We and others have previously shown a descending prostanergic facilitation on spinal nociceptive processing in anaesthetised animals, which has been proposed to exert a tonic influence (Heinricher *et al*. 2004; Leith *et al*. 2007). It is possible that these facilitatory influences could be induced by the experimental surgery, and might not be present in the normal animal (Vanegas & Schaible, 2004). To exclude this possibility, and to aid in the interpretation of data on descending modulation of dorsal horn neuronal responses, we first determined whether tonic COX-dependent facilitation was apparent in awake naïve rats, as well as in anaesthetised rats. Injection of the non-selective COX inhibitor ketoprofen into PAG resulted in a significant increase in thermal withdrawal threshold not found with vehicle injection (Fig.1; Kruskal–Wallis statistic 18.45, *P* = 0.0004), showing that there was demonstrable descending prostanergic facilitation in the absence of anaesthesia. These experiments were designed to provide proof of concept (i.e. that there was a COX-dependent mechanism that was not activated by surgical procedures) and, as such, a non-selective COX inhibitor was used. Once established, the dorsal horn studies used a COX-1 antagonist based on previous findings (Leith *et al*. 2007).

### Responses of neurons to peripheral stimuli

Recordings were made from 27 deep dorsal horn neurons from 26 rats, with a mean depth of 779 ± 31 μm from the cord surface (Table1), to characterise responses to peripheral heat ramps. All neurons responded to both innocuous and noxious stimuli (summarised in Table1), and were therefore WDR or class 2 neurons (Menetrey *et al*. 1977, 1979). All neurons had ERFs located on the dorsal surface of the left hindpaw, with some extending into the toe region and/or into the lateral plantar surface, and typically covering an area equivalent to one-quarter of the dorsal surface of the hindpaw.

**Table 1:** Summary of characteristics and response properties of recorded units

	Parameter	Mean ± SEM	Median	Range
Depth (μm)		779 ± 31	760	510–1170
Spontaneous activity (spikes s^−1^)		2.9 ± 0.5	2.0	0–15.0
Thermal stimulation				
A-nociceptor activation	Overall response (spikes per ramp s^−1^)	24.7 ± 2.3	24.1	4.5–56.2
	Firing threshold (°C)	44.8 ± 0.5	44.6	38.0–50.4
	Suprathreshold activity (spikes s^−1^)	40.0 ± 2.3	39.7	13.4–78.2
C-nociceptor activation	Overall response (spikes per ramp s^−1^)	14.3 ± 2.0	10.9	0.4–34.1
	Firing threshold (°C)	42.6 ± 0.8	41.7	36.9–52.5
	Suprathreshold activity (spikes s^−1^)	21.8 ± 1.4	21.3	3.8–38.3
Electrical stimulation	A-fibre threshold (V)	1.7 ± 0.1	2.0	0.5–3.0
	C-fibre threshold (C+ve cells only, *n* = 26; V)	11.1 ± 0.9	10.5	6.0–25.0
	Spikes at C-fibre latency at 1.5 T (C+ve cells only, *n* = 26)	4.6 ± 0.6	3.7	0.9–10.9
	Spikes at C-fibre latency at 3 T (C+ve cells only, *n* = 26)	6.7 ± 0.8	4.9	2.1–15.1

The data show the depth of neurons (*n* = 27) from the cord surface, level of spontaneous activity and response properties to electrical and thermal stimulation of recorded units.

Control responses to fast and slow heat ramps, which preferentially activate A- and C-fibre heat nociceptors, respectively, were examined in all 27 neurons. The degree of C-nociceptor input and its relationship with the neuronal response to slow ramp heat stimulation have been previously reported (Leith *et al*. 2007). A-nociceptor activation evoked responses over the entire duration of the ramp (mean 24.7 ± 2.3 spikes s^−1^), with a mean firing threshold of 44.8 ± 0.5°C and a mean suprathreshold firing rate of 40.0 ± 2.3 spikes s^−1^. Responses to C-nociceptor activation were variable, with some cells responding very vigorously, others only very weakly and one cell did not respond at all, consistent with a variable degree of C-nociceptor input to dorsal horn neurons (Waters & Lumb, 2008). C-nociceptor activation also evoked responses over the entire duration of the ramp (mean 14.3 ± 2.0 spikes s^−1^, *n* = 27, 15.0 ± 2.0 spikes s^−1^ (*n* = 26, excluding the cell that did not respond)), with a mean firing threshold of 42.6 ± 0.8°C and a mean suprathreshold firing rate of 21.8 ± 1.4 spikes s^−1^ (Table1). Both the suprathreshold firing rates (*t*_26_ = 4.82, *P* < 0.0001, *n* = 27 per group) and the response thresholds were significantly higher in response to A-nociceptor compared to C-nociceptor activation (*t*_25_ = 2.97, *P* = 0.0065, *n* = 27 (A-nociceptors) and 26 (C-nociceptors, as one cell did not respond to C-input (C-ve)) (Fig.2).

**Figure 2:**
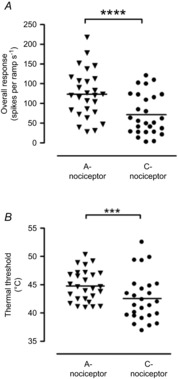
Responses of dorsal horn neurons to thermal activation of A- and C-nociceptors are significantly different *A*, the magnitude of overall responses of class 2 deep dorsal horn neurons to activation of A-fibre nociceptors was greater than that to activation of C-fibre nociceptors. *B*, thermal thresholds for A-nociceptor activation were significantly higher than for C-nociceptor activation. ***P = 0.0065, ****P < 0.0001, paired Student's *t* test.

All neurons responded to electrical stimulation of their ERFs with a short latency (<20 ms, A-nociceptor-mediated response (mean threshold of 1.7 ± 0.1 V)), and in 26/27 neurons a long latency (90–350 ms) C-fibre-mediated response was also observed at higher stimulus intensities (mean threshold 11.1 ± 0.9 V). Stimulus intensities required to evoke activity at C-fibre latency were always significantly higher than those required to evoke activity at A-fibre latency (*P* < 0.001, Wilcoxon signed rank test), suggesting that the long latency responses observed were indeed due to C-fibre activation rather than A-fibre activation via a polysynaptic relay. The magnitude of C-fibre drive to cells was quantified; in the 26 C+ve neurons, the mean number of spikes evoked at C-fibre latency at 1.5 times C-fibre threshold was 4.6 ± 0.6, and was significantly greater at 3 times C-fibre threshold (6.7 ± 0.8 spikes; *P* = 0.0003, Wilcoxon signed rank test). In the C-ve cell, no spikes were evoked at C-fibre latency, even at stimulus intensities up to 90 V (Waters & Lumb, 2008). This cell gave no clear response to slow heat ramp stimulation, but did give a clear and robust response to fast heat ramps.

Based on the number of electrically evoked action potentials at C-fibre latency in cells in this study, a spectrum of C-fibre drive was observed, with some cells responding vigorously to C-fibre stimulation (‘strong’ C+ves), others responding less vigorously (‘weak’ C+ves) and one cell not responding (C-ve). We have previously reported data from these cells showing that those that responded most robustly to slow heat ramps had more vigorous firing to electrically evoked C-fibre input, whereas those that responded only weakly to slow heat ramps in general had relatively weak electrically evoked C-fibre input (see Fig.2; Leith *et al*. 2007). There is a highly significant positive correlation between the electrically evoked C-fibre activity and the overall response to slow heat ramps at both 1.5 and 3 times C-fibre threshold in individual WDR neurons (Leith *et al*. 2007).

### COX-1 inhibition by SC-560 in the VL-PAG increases firing thresholds of dorsal horn neurons to both A- and C-nociceptor activation

As noxious withdrawal reflexes to C-nociceptor stimulation were affected by PAG COX-1 inhibition (Leith *et al*. 2007), we determined the effects of the COX-1 inhibitor SC-560 or vehicle administration into the VL-PAG on dorsal horn neuronal responses in the 18 C+ve cells (Figs 3 and 4). SC-560 increased thresholds to both C-nociceptor (Fig.3*A* and *B*; two-way between-factors ANOVA: effect of drug *F*_1,134_ = 76.8, *P* < 0.0001; effect of time *F*_9,134_ = 2.1, *P* = 0.04; interaction *F*_9,134_ = 2.9, *P* = 0.004) and A-nociceptor activation (Fig.3*C* and *D*; two-way between-factors ANOVA: effect of drug *F*_1,144_ = 91.6, *P* < 0.0001; effect of time *F*_9,144_ = 2.2, *P* = 0.02; interaction *F*_9,144_ = 3.8, *P* = 0.0003). The effect of SC-560 on C-nociceptor peak responses was significantly greater than the effect of vehicle (Fig.4*A*) whereas the overall change was significantly greater than vehicle for both A- and C-nociceptor stimulation. The increase in *peak* threshold caused by SC-560 injection (A-nociceptor: baseline: 44.1 ± 0.9°C; peak 48.1 ± 0.9°C at 57 min: C-nociceptor: baseline: 42.2 ± 1.3°C; peak 46.4 ± 1.1°C at 65 min; one-way ANOVA *F*_3,27_ = 10.90, *P* < 0.0001; Fig.4*A* and *B*)) and the change in *overall* threshold over time by SC-560 (measured as AUC; A-nociceptor: 186.7 ± 25.3 min·°C *vs*. C-nociceptor: 211.2 ± 35.9 min·°C, one-way ANOVA *F*_3,27_ = 3.97, *P* = 0.018) were similar for A- and C-nociceptor stimulation. Vehicle alone had no significant effect on firing thresholds compared to control responses.

**Figure 3:**
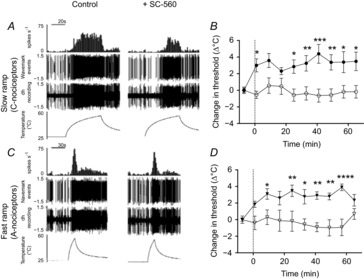
COX-1 inhibition in the VL-PAG results in a significant increase in firing thresholds to both C- and A-nociceptor activation *A*, digitised traces of raw data displaying responses of a moderate C+ve neuron to C-nociceptor activation, before and after administration of SC-560 into the VL-PAG. *B*, SC-560 administration into the VL-PAG resulted in an increase in response threshold of dorsal horn neurons to C-nociceptor activation compared to vehicle. *C*, digitised traces of raw data displaying responses of a moderate C+ve neuron to A–nociceptor activation, before and after administration of SC-560 into the VL-PAG. *D*, SC-560 administration into the VL-PAG resulted in an increase in response threshold of dorsal horn neurons to A-nociceptor activation compared to vehicle. Data are mean ± SEM. *P < 0.05, **P < 0.01, ***P < 0.001, ****P < 0.0001, two-way ANOVA plus *post hoc* Bonferroni tests.

**Figure 4:**
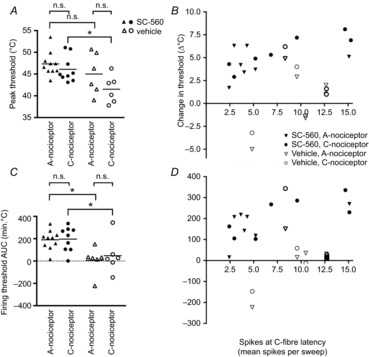
PAG administration of SC-560 results in a significant change in firing thresholds of both A- and C-nociceptors The level of threshold change produced by PAG SC-560 was not significantly different between A- and C-nociceptors, and was significantly correlated with the degree of C-nociceptor input to the cells. *A*, peak change in firing thresholds to A- and C-nociceptor activation produced by SC-560 and vehicle administration into the VL-PAG. *B*, correlation between peak threshold change and the magnitude of C-nociceptor input to each cell evoked by electrical stimulation at 3 times C-fibre threshold (*r* = 0.6, *n* = 14, *P* = 0.03, Spearman's correlation). *C*, overall change with time (AUC) of firing thresholds to A- and C-nociceptor activation produced by SC-560 and vehicle administration into the VL-PAG. *D*, correlation between overall threshold change with time and the magnitude of C-nociceptor input to each cell evoked by electrical stimulation at 3 times C-fibre threshold (*r* = 0.7, *n* = 14, *P* = 0.007, Spearman's correlation). *P < 0.05; n.s., not significant by one-way ANOVA plus Bonferroni *post hoc* tests.

Given that we previously described a mean inhibition of noxious pinch-evoked responses in C+ve neurons, but a mean facilitation of C-ve neurons following non-specific neuronal activation of the PAG (Waters & Lumb, 2008), we hypothesised that the overall magnitude of inhibition (raised threshold) of C+ve neurons evoked by COX-1 inhibition might also be related to the profile of their afferent drives, in particular to the level of C-fibre drive. There was a significant positive correlation between the magnitude of change in both peak and overall change in threshold with time (AUC) in response to SC-560 and the magnitude of C-fibre drive at both 3 times threshold stimulation (Fig.4*B* and *D* filled symbols; threshold change, *r* = 0.6, *n* = 14, *P* = 0.009; overall (AUC) inhibition, *r* = 0.7, *n* = 14, *P* = 0.007; note that in three cells treated with drug, electrically evoked activity at 3 times threshold was not determined, and these are therefore not included), and 1.5 times threshold (data not shown). These data indicate that those cells with greater C-fibre drive were indeed subject to greater descending control from the PAG. Neither variable correlated with the response to vehicle (Fig.4*B* and *D*, open symbols: threshold change, *r* = 0.04, *n* = 10, *P* = 0.9; overall change in threshold with time, *r* = 0.12, *n* = 10, *P* = 0.7).

### Encoding of C-fibre information is lost, whereas encoding of A-fibre information remains intact following COX-1 inhibition in the VL-PAG

Neuronal activity evoked by A-nociceptor activation increased linearly with increased skin temperature above threshold and was best modelled by a linear regression line (Fig.5*A*; slope 3.61 ± 0.36 spikes s^−1^·°C, *y*-axis intercept 21.0 ± 2.4 spikes s^−1^). There was a positive correlation between A-nociceptor-evoked activity and contact skin temperature (*r* = 0.99, *P* < 0.0001, Spearman's rank correlation). In contrast, neuronal activity evoked by C-nociceptor activation increased linearly with temperature over approximately the first 5°C above threshold, then reached a plateau and subsequently decreased as skin temperature increased further.

**Figure 5:**
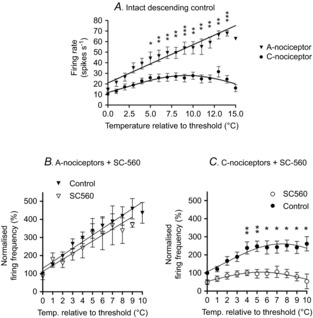
Temperature encoding of A-nociceptor information is maintained in the face of loss of prostanergic descending control from the PAG following SC-560 administration, whereas encoding of C-nociceptor information is lost *A*, temperature–response relationships to A-nociceptor (▾, *n* = 10; and ▽, *n* = 16) and C-nociceptor (○, *n* = 9; and •, *n* = 14) rates of skin heating, showing that the ability of cells to encode temperature is significantly different between fast and slow heat ramp-evoked responses under normal circumstances. Raw unit activity expressed as mean ± SEM is shown with linear and non-linear (Gaussian) regression lines modelling A-nociceptor- and C-nociceptor-evoked activity, respectively. *B*, normalised temperature–response relationships to A-nociceptor activation before and after SC-560 administration into the VL-PAG. *C*, normalised temperature–response relationships to C-nociceptor activation before and after SC-560 administration into the VL-PAG. The encoding functions denoted by the non-linear regression lines for C-nociceptors were significantly different after intra-VL-PAG injection (*P* < 0.0001, F test), whereas the A-nociceptor encoding function was unchanged (*P* = 0.32, F test). Data are expressed as mean ± SEM, with the regression lines for the data also shown. Note that in *B* the individual regression lines are shown although the comparison of the data shows that a single linear regression line fits both data sets, i.e. there is no difference between the data sets before and after SC-560. Individual points are also shown where significantly different between conditions. *P < 0.05, **P < 0.01, ***P < 0.001; two-way ANOVA followed by Bonferroni *post hoc* test. A-nociceptors: effect of drug *F*_1,226_ = 4.6, *P* = 0.03, effect of temperature relative to threshold *F*_10,226_ = 8, *P* < 0.0001, no interaction *F*_10,226_ = 0.4, *P* = 0.9. C-nociceptors: effect of drug *F*_1,195_ = 84.3, *P* < 0.0001, effect of temperature relative to threshold *F*_10,195_ = 2.3, *P* = 0.01, no interaction *F*_10,195_ = 1.2, *P* = 0.3.

There was a significant difference between A- and C-nociceptor encoding functions when linear *vs*. Gaussian fits were compared (A-nociceptors – linear (*F*_1,164_ = 2.52, *P* = 0.11) *vs*. C-nociceptors – Gaussian (*F*_1,138_ = 6.8, *P* = 0.009)), i.e. the A- and C-nociceptor response functions could not be fit by the same linear function. C-nociceptor-evoked activity was better modelled by a Gaussian distribution (*r*^2^ = 0.22) than by linear regression (*r*^2^ = 0.13). In C-nociceptors, evoked activity did not correlate significantly with temperature (*r* = 0.43, *P* = 0.10; Spearman's rank correlation). A-nociceptor activation generally evoked higher rates of dorsal horn neuronal activity at any given temperature than C-nociceptor activation (Fig.5*A*). Comparison of the lines of best fit for the encoding functions showed that linear temperature encoding of A-nociceptor-evoked activity remained unaltered after administration of SC-560 into the PAG (Fig.5*B*: *F*_2,242_ = 1.16, *P* = 0.32). However, the Gaussian temperature encoding of C-nociceptor-evoked activity was significantly reduced by intra-PAG SC-560 injection (Fig.5*C*; *F*_3,211_ = 31.7, *P* < 0.0001).

### Magnitude of SC-560 inhibition of noxious neuronal responses is independent of PAG injection site

A possible explanation for variability in the magnitude of descending control is the difference in the injection sites within the VL-PAG between experiments. To investigate whether the level of descending inhibition was related to injection site within the PAG, the rostrocaudal coordinates of each site were plotted against the overall inhibition of neuronal firing threshold (Fig.6). There was no obvious relationship between the level of descending inhibition and injection site (for A- and C-nociceptor activation combined: *r* = 0.05, *P* = 0.82, *n* = 21; A-nociceptor alone: *r* = 0.04, *n* = 11, *P* = 0.96; C-nociceptor alone: *r* = 0.19, *n* = 10, *P* = 0.59; Spearman's correlation).

**Figure 6:**
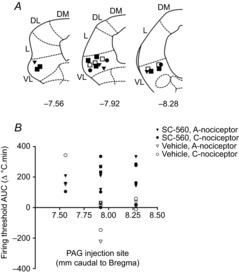
Rostrocaudal site of injection of SC-560 within the PAG does not influence the degree of inhibition of A- or C-nociceptor-evoked responses *A*, injection sites within the VL-PAG: sites of SC-560 injection tested on slow ramps only (•), fast only (▾), or both (▪); vehicle sites tested on both fast and slow heat ramps (□); coordinates are relative to Bregma (DM, dorsomedial; DL, dorsolateral; L, lateral; VL, ventrolateral). *B*, there was no correlation between the rostrocaudal location of the injection sites within the PAG and the overall change over time (AUC) in neuronal firing thresholds to A- and C-nociceptor activation produced by intra-VL-PAG injection of SC-560 (*r* = 0.23, *n* = 19, *P* = 0.33; Spearman's correlation) or vehicle (*r* = −0.3, *n* = 12, *P* = 0.2). Note that some injection sites are overlapping.

## Discussion

A- and C-fibre nociceptors have distinct and independent contributions to the perception of pain quality, and the development of chronic pain, as increased C-nociceptor drive after damage sensitises spinal circuits to A-nociceptor inputs (Treede & Magerl, 2000; You *et al*. 2010). There is evidence to suggest a separation in the central processing of information arising from A- *vs*. C-nociceptors, including differential descending modulation (McMullan & Lumb, 2006a,*b*2006b; Koutsikou *et al*. 2007; Leith *et al*. 2007; You *et al*. 2010). However, the mechanisms underlying this differential modulation remain unknown. We demonstrate the presence of a tonic COX-dependent descending control system on spinal nociceptive processing from VL-PAG present in awake normal animals. In dorsal horn WDR neurons, the extent of descending facilitation is greater for neurons receiving a greater C-nociceptor drive, consistent with our previous findings that COX-1 inhibition had much greater effects on noxious C-nociceptor withdrawal reflexes (Leith *et al*. 2007). By contrast, the temperature encoding in response to A- and C-nociceptive inputs was differentially modulated by this descending system.

### Spinal processing of A- *vs*. C-fibre nociceptive information

Not all deep dorsal horn neurons receive C-nociceptor drive – reports suggest that 51–88% of dorsal horn neurons are C+ve depending on species (rat and cat, lower end of range from cat; Gregor & Zimmermann, 1972; Menetrey *et al*. 1977; Waters & Lumb, 2008). Cells in deep laminae receive C-nociceptor input either indirectly through interneurons in lamina I (which were not studied here), or directly through WDR dendrites extending into superficial laminae. We found 26/27 neurons to be C+ve, but stimulation site (percutaneous *vs*. nerve trunk) and search stimulus (pinch *vs*. heat) may account for these differences. Our descriptions of the response characteristics of dorsal horn neurons to C- *vs*. A-nociceptor activation are consistent with those of dorsal horn WDR neurons and primary afferents reported elsewhere (Adriaensen *et al*. 1983; Handwerker *et al*. 1991; Tillman *et al*. 1995a,*b*1995b; Yeomans & Proudfit, 1996; Treede *et al*. 1998; McMullan & Lumb, 2006b), in that (a) C+ve WDR neurons are driven by both fast and slow ramps, and (b) the evoked firing patterns and temperature encoding properties are distinct and different. A-nociceptors show steep, linear temperature–response relationships with greater overall firing rates, whereas C-nociceptors exhibit more Gaussian temperature–response relationships, with lower total responses, that plateau at higher stimulus intensities (Garell *et al*. 1996; Andrew & Greenspan, 1999; Slugg *et al*. 2000). The plateau in dorsal horn neuronal encoding of C-nociceptor input could possibly be attributed to peripheral desensitisation of TRPV1 in the afferents activated during the stimulus (McMullan *et al*. 2004; Joseph *et al*. 2013), and similar patterns in mechanical (as opposed to thermal) stimulus encoding properties have also been reported in mechanoheat-responsive C-nociceptors (Slugg *et al*. 2000). This implies that these encoding functions are inherent properties of the peripheral nociceptors rather than an artefact of the stimulation protocol. These findings indicate that A-fibre nociceptors encode spatial and intensity components of noxious stimulation more accurately than C-fibre nociceptors. That the same response properties are observed in nociceptive afferents and dorsal horn neurons indicates that the temporal patterns of information transmitted to higher centres may remain highly distinct and are dependent on the fibre types activated by the peripheral stimulus.

### Differential descending control of spinal A- and C-nociceptive processing by COX-1-sensitive mechanisms in the PAG

Inhibition of COX-1 and disruption of descending facilitation from the VL-PAG produced complex anti-nociceptive effects on neuronal activity in the dorsal horn. Loss of descending facilitation affected overall responses to both A- and C-nociceptor stimulation, which were increased to a similar extent in individual C+ve WDR deep dorsal horn neurons and remained elevated over a similar time course. This is consistent with our previous study (McMullan & Lumb, 2006a), in which non-specific activation of the DL-PAG was used. Similarly, non-selective COX inhibition in the PAG depressed deep dorsal horn neuronal activity to electrical C-fibre stimulation and noxious pinch (a stimulus likely to co-activate A- and C-fibres; Carlsson & Jurna, 1987; Vanegas *et al*. 1997). The pharmacological inhibition of COX-1 is known to allow diversion of arachidonic acid metabolism to other pathways, and thus lead to the production of other lipid mediators. Our findings might therefore reflect an increase in hydroperoxyeicosatetraenoic acid and leukotrienes, lipoxins or endocanabinoids (Bari *et al*. 2011), some of which are known to exert anti-nociceptive effects in the PAG (Palazzo *et al*. 2008; de Novellis *et al*. 2012), rather than a decrease of a tonic cyclooxygenase activity.

The prevailing view is that the PAG exerts indiscriminate modulatory effects on nociceptor-evoked responses in spinal dorsal horn neurons. Here, we show that the VL-PAG has the capacity to exert a *highly selective* tonic prostanergic facilitation of C+ve dorsal horn neurons, in the normal animal. This is manifest in two ways: first, strongly C+ve neurons are subject to greater levels of descending control than weak C+ve neurons. This is the case whether such control is facilitatory or inhibitory, as we have also shown with non-specific chemical activation of the PAG where responses of WDR neurons with both A- and C-fibre inputs (C+ves) were inhibited, whereas neurons with A-fibre drive alone (C-ves) were facilitated (Waters & Lumb, 2008). Secondly, in contrast to the relatively non-selective effects of COX-1 inhibition in the PAG on A- and C-nociceptor thresholds, the temperature-response functions of these different nociceptor groups were differentially modulated by loss of facilitation from the VL-PAG. This is likely to be a distinct effect on C-nociceptor inputs rather than an effect on neurons with greater overall input, as the effect on dorsal horn encoding properties was clearly limited to C-nociceptor responses with no effect on those driven by A-nociceptors (Fig.5). The results of the current study provide further insights into the balances of descending control mechanisms that underlie the differential modulation of A- and C-nociceptor inputs into the spinal cord (Vanegas & Schaible, 2004). We can draw no conclusions relating to the descending controls on C-ve neurons due to the small number identified in this study. However, given the relationship between the magnitude of facilitatory effect and C-fibre drive, they would be predicted to be unaffected or possibly facilitated by intra-VL-PAG inhibition of COX-1, as also suggested by the findings of Waters and Lumb (2008).

Our current findings together with our previous studies (McMullan & Lumb, 2006b; Waters & Lumb, 2008) point to a complex interaction of descending modulatory pathways with spinal neurons, which are likely to interact on multisynaptic spinal pathways postsynaptic to the primary afferent terminals (thereby inhibiting both A- and C-fibre afferent input similarly) but presynaptic to the deep dorsal horn neurons studied (leaving spontaneous, and presumably also low threshold, inputs unaffected) (Waters & Lumb, 1997).

### Functional significance

The anti-nociceptive effect observed following COX-1-dependent loss of descending facilitation indicates that COX-1 is tonically active in the VL-PAG in the normal animal, and that this pathway targets the processing of nociceptive input to lamina IV–V WDR neurons. The rise in response threshold was greatest on dorsal horn neurons with strong C-fibre drive, suggesting that these neurons, which are key to the process of central sensitisation and the establishment of chronic pain (Treede & Magerl, 2000), are subject to significant descending facilitation under normal circumstances. Inhibition of a tonic prostanergic descending facilitation on C+ve neurons would remove the ability to encode the dull, diffuse input deriving from C-nociceptor activation while accurate information on the intensity and the rate of rise of the thermal stimulus derived from A-nociceptor activation would remain intact (see also McMullan & Lumb, 2006b).

These data indicate that COX activity in the PAG has an important role in regulating nociceptive processing, setting the gain at the spinal sensory neuronal level, by determining the excitability of dorsal horn neurons to incoming information and balancing the level of A- *vs*. C-nociceptive input transmitted to higher centres. Endogenous prostanergic descending control from the PAG could effectively modulate contrast within the cord, as changes in the strength of the pathway would sharpen some inputs and damp down others through the targeting of different populations of neurons that encode incoming information.

We speculate that, physiologically, this fine balance of descending control over A- *vs*. C-nociceptors might be rapidly altered in different behavioural states and, in so doing, forward information that triggers appropriate, and co-ordinated, behavioural, affective and autonomic responses. For instance, we speculate that decreasing the level of descending facilitation from the PAG would decrease the level of nociceptive information originating from C-fibre afferents from reaching higher structures (distracting information that might impede survival) while maintaining the transmission of rapidly conducted A-fibre-mediated inputs. The latter is predicted to provide a protective function in driving withdrawal responses and directing motivational behaviours that aid survival (e.g. Waters & Lumb, 2008). Additionally, use of centrally penetrant COX inhibitors in acute pain might be expected to have some effect on the more debilitating C-nociceptor-evoked pain.

In addition to their different roles in acute nociceptive processing (slow *vs*. fast pain), C- and A-nociceptor sub-types play distinct roles in chronic pain states (Fuchs *et al*. 2000; Magerl *et al*. 2001; You *et al*. 2010). It is apparent that pathological alterations in descending controls contribute to the development and maintenance of chronic pain states (Porreca *et al*. 2002; Vanegas & Schaible, 2004; Geranton *et al*. 2008; Hu *et al*. 2009), and that these involve intra-PAG prostaglandins (Palazzo *et al*. 2011), and so, as previously suggested (You *et al*. 2010), an understanding of the mechanisms underlying the preferential control of these distinct inputs is both clinically relevant and important.

## Additional information

### Author's present address

Alex W. Wilson: Maccine Pte Ltd, 10 Science Park Road #01-05, The Alpha Singapore Science Park II, Singapore 117684.

## Key points

- A- and C-nociceptors make different contributions to pain. They signal fast and slow pain, respectively, and C-nociceptors drive central sensitisation.
  Cyclooxygenase-1 (COX-1)-dependent descending facilitation from the periaqueductal grey (PAG) affects C-nociceptor spinal reflexes, but leaves A-nociceptor reflexes largely untouched.
- We studied the responses of spinal wide dynamic-range dorsal horn neurons to A- and C-nociceptor stimulation before and after COX-1 inhibition in the PAG.
- Inhibition of COX-1 descending facilitation disrupted the neuronal encoding of C-nociceptor information, but left the encoding of A-nociceptor information untouched.
- Inhibition of COX-1-dependent descending facilitation increased the activation threshold of wide dynamic range neurons to both A- and C-nociceptor input.
- The COX-dependent descending system from the PAG can therefore control the amount of C-nociceptor information reaching the CNS (slow, burning, aching, debilitating pain) without affecting the protective A-nociceptor information (sharp, well-localised, protective pain).

## Abbreviations

AUC: area under the curve
COX-1: cyclooxygenase-1
ERF: excitatory receptive field
PAG: periaqueductal grey
PGE2: prostaglandin E2
WDR: wide dynamic range
